# The imbalance of gut microbiota and its correlation with plasma inflammatory cytokines in pemphigus vulgaris patients

**DOI:** 10.1111/sji.12799

**Published:** 2019-07-08

**Authors:** Shuli Huang, Jing Mao, Lin Zhou, Xia Xiong, Yongqiong Deng

**Affiliations:** ^1^ Department of Dermatology & STD The Affiliated Hospital of Southwest Medical University Luzhou China

**Keywords:** cytokines, gut microbiota, pemphigus vulgaris

## Abstract

Pemphigus vulgaris (PV) is an autoimmune disease characterized by the production of IgG autoantibodies owing to an imbalance in the Th1/Th2 and Th17/Tregs cell pathways. The role of gut microbiota in the development of immune system and autoimmune diseases has been unraveled in the last two decades. However, data pertaining to gut microbiota of PV patients is largely lacking. We aimed to compare the gut microbiota of PV patients and healthy controls and assessed potential correlation with circulating cytokines of Th1/Th2/Th17 cell. Faecal bacterial diversity was analysed in 18 PV patients and 14 age‐ and gender‐matched healthy individuals using hypervariable tag sequencing of the V3‐V4 region of the 16S rRNA gene. Plasma levels of 20 inflammatory cytokines were assessed using the Luminex screening system. As a result, we identified 10 differentially abundant taxa between patients and controls. At the genera level, *Lachnospiracea_incertae_sedis* and *Coprococcus* decreased, while *Granulicatella*, *Flavonifractor* enriched in PV. Plasma levels of C5a, interleukin (IL)‐2R, IL‐6, IL‐8, IL‐7, IL‐1β, IL17A, IL‐5 and IL‐21 were significantly increased in PV *Flavonifractor* exhibited a positive correlation with C5a, IL‐6, IL‐8, IL‐7, IL‐1β, IL17A and IL‐21. *Lachnospiracea_incertae_sedis* and *Coprococcus* showed a negative correlation with IL‐17A. Our results are consistent with the hypothesis that PV patients have gut microbial dysbiosis which might contribute to the immune disorder and the development of PV.

## 1 INTRODUCTION

Pemphigus vulgaris (PV) is a life‐threatening autoimmune blistering disease clinically characterized by intra‐epidermal blisters and acantholysis resulting from the formation of IgG autoantibodies against desmoglein (Dsg)3, which impairs epidermal cell‐cell adhesion.^1^ Despite recent advances in the diagnosis and treatment of PV, the mortality rate of patients approaches 6%; moreover, several gaps persist in our understanding of the pathogenesis of this disease.^2^, ^3^ Early studies focused on the role of humoral immune responses in the development of the disease, and more recent evidence further suggests that production of autoantibodies due to an imbalance in the Dsg3‐sensitized Th1 and Th2 cell pathways plays a key role in the pathogenesis of PV.^4^, ^5^ A number of recent studies have examined T cell and cytokine profiles in pemphigus patients with the hopes of clarifying the complex pathogenesis of this rare disease. Unfortunately, results have varied. Although a recent article reported higher serum levels of inflammatory Th1/Th17 cytokines in PV patients, most of studies proven to be a Th2 predominant response, along with concomitant suppression of Th1 response.^6^, ^7^ In addition, studies also presented the possibility of involvement of the unbalanced Th17/Tregs cells in the development and progression of autoimmune bullous diseases.^8^ Production of pro‐inflammatory cytokine production [especially, tumour necrosis factor (TNF)‐α, IL‐1 α and IL‐8] has also been implicated in the maintenance of pathogenic mechanisms of PV.^9^ While the contribution of T cell and cytokines in pemphigus is clear, the precise mechanisms by which these cytokines induce blister formation are not completely understood.

Additionally, the global incidence of autoimmune diseases is estimated at 3%‐5%, with an increasing trend observed over the past half‐century.^10^ Although multiple genetic factors are implicated in the development of these disorders, genetic drift alone cannot explain all phenomena involved in the pathogenesis of these diseases. The relationship between environmental factors especially gut microbiota and the increased prevalence of autoimmune disorders such as lupus erythematosus (SLE), rheumatoid arthriti, psoriasis, systemic sclerosis and Behcets disease has evoked considerable attention.^11^, ^12^, ^13^, ^14^, ^15^ The immune system co‐evolves with the microbiota with a complex interaction between the two. While the immune system affects microbial inhabitation and activity, the microbiota, in turn, modulates the innate and adaptive immune mechanisms, including peripheral differentiation of Th cells.^16^, ^17^, ^18^ For example, gut *segmented filamentous bacteria* (SFB) were shown to promote a Th17 response and autoimmunity in mouse models of arthritis and multiple sclerosis.^11^ In another study, high‐salt diet was shown to enhance the expression of pro‐inflammatory genes and suppress several anti‐inflammatory cytokines and chemokine genes, via reduction in the gut *Lactobacillus* sp. and protective short‐chain fatty acid production.^19^

Based on the above findings, we hypothesized that T cell and the associated cytokines may promote the pathogenesis of PV via interaction with the dysbiosis of gut microbiota. This study was designed to investigate the gut microbiome in faecal samples from PV patients and healthy controls. Circulating pro‐inflammatory markers and Th1/Th2/Th17/Treg‐related cytokines and chemokines were also assessed. This is the first study to report the dysbiosis of gut microbiota and to demonstrate the association between microbiome and cytokines in PV.

## 2 MATERIALS AND METHODS

### 2.1 Patients

Consecutive patients from the Affiliated Hospital of Southwest Medical University between January 2017 and May 2018 were screened for the presence of PV. After obtaining informed consent, skin biopsy for histopathology and direct immunofluorescence (DIF) examination were performed. The phenotype of PV, body mass index (BMI), age at onset, multiplicity of mucosal involvement, and relapse and remission rates were also recorded and reviewed. All PV patients who qualified the study criteria were offered enrolment. Age‐ and gender‐matched healthy subjects were enrolled as controls.

This research was approved by the Institutional Review Board of The Affiliated Hospital of Southwest Medical University. Written informed consent was obtained from all participants prior to their enrolment.

### 2.2 Inclusion and exclusion criteria

Patients with recent‐onset PV who had active PV skin lesions were prospectively enrolled. The diagnosis was based on clinical manifestations, histopathological evaluation and direct immunofluorescence.^20^ The inclusion criteria were age ≥18 years and BMI between 18 and 25 kg/m^2^.

The exclusion criteria were as follows: age <18 years old; BMI ≥25 or BMI <18; known history of chronic disease such as chronic dermatosis (eg psoriasis, seborrhoeic dermatitis, contact dermatitis, atopic dermatitis and pruritus), autoimmune or rheumatic diseases, metabolic diseases, chronic gastrointestinal diseases and malignant tumours; pregnant or breastfeeding women; presence of acute infection; history of gastrointestinal tract surgery; recent (<6 months) use of antibiotic or consumption of probiotics.

### 2.3 Sample collection

Fresh stool samples from each participant were collected in a sterile container, immediately homogenized, divided into ten aliquots of 220 mg, and frozen at −80°C within 30 minutes. An average of 2 mL of peripheral blood was collected from each participant into an anticoagulant tube. Plasma samples were obtained by centrifugation at 3000 rpm for 15 minutes and stored at −80°C. During the period of sample collection, specimens were kept at 4°C.

### 2.4 Detection of cytokines

Twenty‐one cytokines and chemokines (C5a, YKL‐40, IP‐10, CD163, IL‐2R, Osteopontin, ITAC‐1, TNF‐alpha, IL‐6, IL‐8/CXCL8, IL‐7, IL‐10, IL‐1beta, IFN‐gamma, IL‐4, IL‐17A, IL‐2, IL‐13, IL‐5, IL‐21, IL‐23) were tested by Human Magnetic Luminex Screening Assay (LXSAHM‐05P3/LXSAHM‐14P1, R&D Systems, Inc). Plasma levels of cytokines were determined using a Luminex 200 System (EMD Millipore) according to the manufacturer's instructions. The coefficient of variation between the duplicate wells was controlled within 10%, and R2 of the standard curve was at least 0.999.

### 2.5 DNA extraction, PCR amplification and sequencing

Microbial DNA was extracted from faecal samples using the QIAamp DNA Stool Minikit (Qiagen Ltd) according to manufacturer's protocols. The V3‐V4 region of the bacteria 16S ribosomal RNA genes were amplified by PCR using primers 341F 5’‐CCTACGGGRSGCAGCAG)‐3’ and 806R 5’‐GGACTACVVGGGTATCTAATC‐3. We used KAPA HiFi Hotstart ReadyMix PCR kit for high‐fidelity amplification and NanoDrop 2000 spectrophotometer and 2% agarose gel electrophoresis for assessing the quality of amplicons. After preparation of library, these tags were sequenced on Illumina Hiseq platform (Illumina, Inc) for paired end reads of 250 bp, which were overlapped on their 3 ends for concatenation into original longer tags. DNA extraction, library construction and sequencing were conducted at the Realbio Genomics Institute (Shanghai, China).

### 2.6 Sequence‐based microbiota analysis and statistical analysis

Paired End Reads were spliced through Overlap relationship between Reads after sequencing by Illumina platform. Spliced Reads were further checked with respect to their rest lengths and average base quality to obtain Clean Reads. 16S sequences were restricted between 220 and 500 bp such that the average Phred score of bases was no worse than 20 (Q20) and no more than 3 ambiguous N. Sequences were grouped into operational taxonomic units (OTUs) using the average neighbour algorithm; only the sequences with frequency >1, which tend to be more reliable, were clustered into OTUs, each of which had a representative sequence. OTUs were clustered with 97% similarity using UPARSE (http://drive5.com/uparse/), and chimeric sequences were identified and removed using USEARCH (version 7.0). Each representative tags was assigned to a taxa by RDP Classifier (http://rdp.cme.msu.edu/) against the RDP database (http://rdp.cme.msu.edu/) using a confidence threshold of 0.8.

OTU profiling table and alpha/beta diversity analyses were also performed using the python scripts of QIIME, while rarefaction curves were made. Each sample was randomized with sufficient sequencing depth to avoid deviations in the analysis due to different sample sizes; Alpha Diversity Index was used to measure the sequence depth. Principal Coordinates Analysis (PCoA) of faecal samples based on 16S rRNA sequences using both unweighted and weighted UniFrac to measure Beta Diversity, which was performed on the resulting matrix of distances between each pair of samples. LefSe analysis (LDA EffectSize) based on linear discriminant analysis (LDA) was applied to demonstrate differential abundance of bacterial taxa among the two groups. Only those taxa that achieved a log LDA score >2 were ultimately considered and verified by Wilcoxon test and Kruskal test using R3.1.0. Person correlation analysis was performed to assess the correlation between gut microbes and cytokines.

All P‐values reported are two‐sided, and *P* < 0.05 was considered to be statistically significant. We also applied the Benjamini and Hochberg false discovery rate test (FDR) or calculated the 95% confidence intervals (CI), if the FDR *q* value was >0.1.

### 2.7 Analysis of predicted metagenomes

PICRUSt was applied to analyse both 16S rRNA gene relative abundances and the predicted metabolic data. The protein sequences of genes in the merged gene catalogue were aligned to the Kyoto Encyclopedia of Genes and Genomes(KEGG) bioinformatics database (8th KEGG release, December 2014). The Statistical Analysis of Metagenomic Profiles (STAMP) software was used for data filtering and statistical analyses.

## 3 RESULTS

### 3.1 Patient characteristics

Eighteen patients with active PV and 14 age‐ and gender‐matched healthy controls were included in the study. The background characteristics are summarized in Table 1. All participants had a BMI <25 kg/m^2^. The clinical phenotype of PV patients was muco‐cutaneous (n = 10; 55.6%) and isolated cutaneous disease (n = 8; 44.4%). None of the patients had isolated mucosal disease. Half of the patients were treatment‐naive while others had received glucocorticoid and/or immunosuppressive therapy in the immediately preceding 3 months.

**Table 1:** Background of PV patients and healthy controls

Factor	PV (n = 18)	CN (n = 14)	*P*‐value
Gender (Female, n, (%))	9 (50%)	5 (35.7%)	
Age (y, mean ± SD)	45.78 ± 13.45	44.57 ± 14.72	0.811
BMI (kg/m^2^)	22.21 ± 2.26	22.46 ± 1.85	0.732
Treatment history (untreated, n (%))	9 (50%)		
Clinical phenotype, n (%)			
Isolated mucosal	0		
Muco‐cutaneous	10 (55.6%)		
Isolated cutaneous	8 (44.4%)		

### 3.2 Diversity and structure of gut microbiota in PV patients and healthy controls

A total of 32 faecal samples were collected from patients and healthy controls for sequencing. Using the relative abundance in each sample of 97% identity (ID) OTU, a total of 607 OTUs were analysed. Among these, 418 OTUs were shared by the PV and control groups. PV samples had special 107 OUTs while control samples had 82 notable OTUs. Based on the results, the core bacteria identified in the study were Blautia, Baceroides, Escherichia/Shigella, Lachnospiraceae and Faecalibacterium.

**Figure 1:**
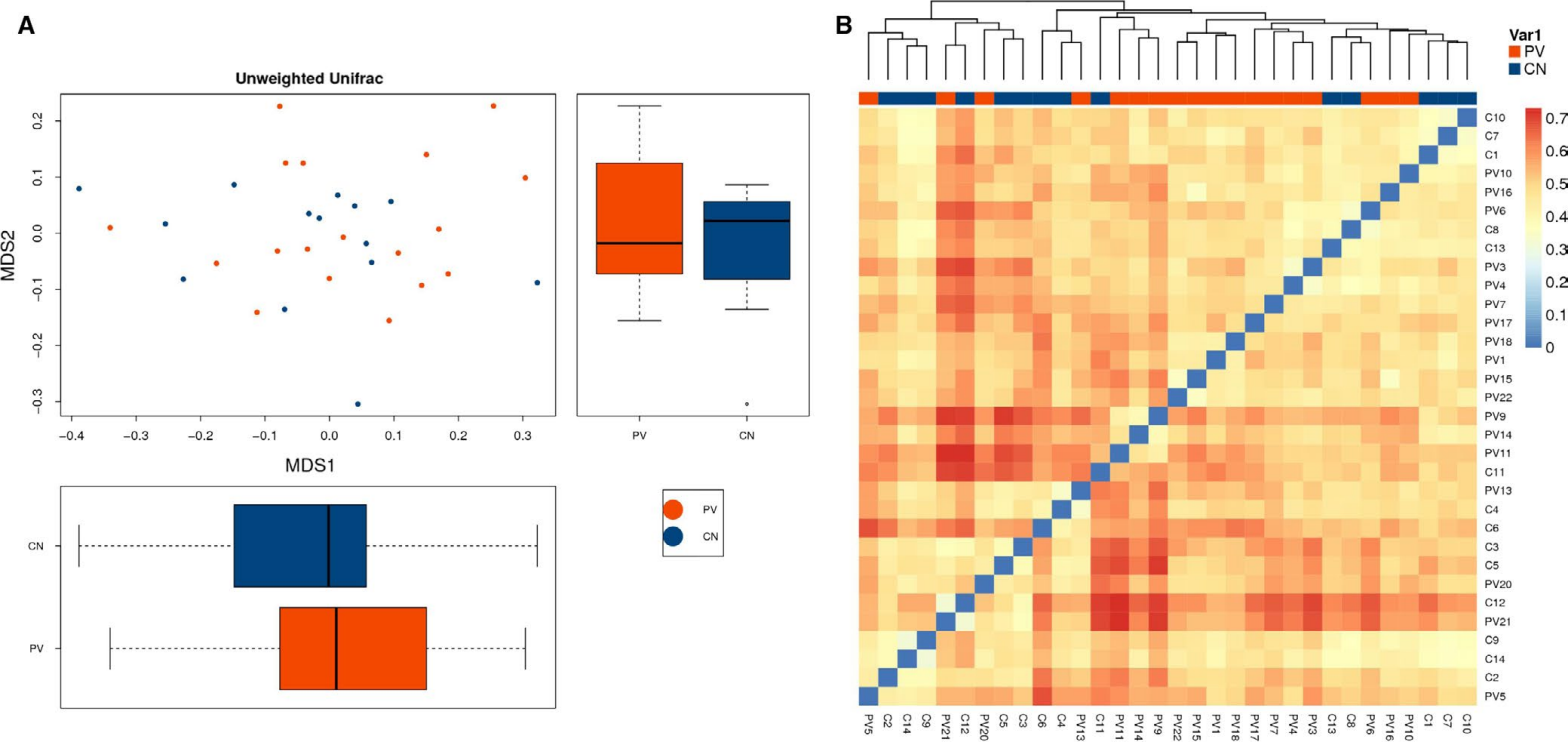
The structure of gut microbiota in PV patients and healthy controls. A, The microbiota of patients with pemphigus vulgaris differed from those of healthy controls based on OTUs with different relative abundances in a UniFrac PCoA plot. B, Heat map was applied to evaluate the similarity of each sample composition

There was no difference between patients and healthy controls with respect to gut microbiota diversity, as assessed by the Shannon diversity index (*P* = 0.72) and Simpson diversity index (*P* = 0.87) (Figure S1). When PCoA analysis was applied to assess discrepancies based on OTUs with different relative abundances, samples from healthy controls clustered in an intermediate position in a UniFrac PCoA plot but the pemphigus vulgaris individuals clustered in a disperse position (Figure 1A). UniFrac phylogenetic distance of the microbe composition among subjects was calculated to investigate the different structures of gut microbes between groups. MRPP analysis indicated a significant difference between PV and control samples (*P* = 0.051). Heat maps also showed different sample composition between the two groups (Figure 1B).

### 3.3 Bacterial taxa difference between PV patients and healthy controls

**Figure 2:**
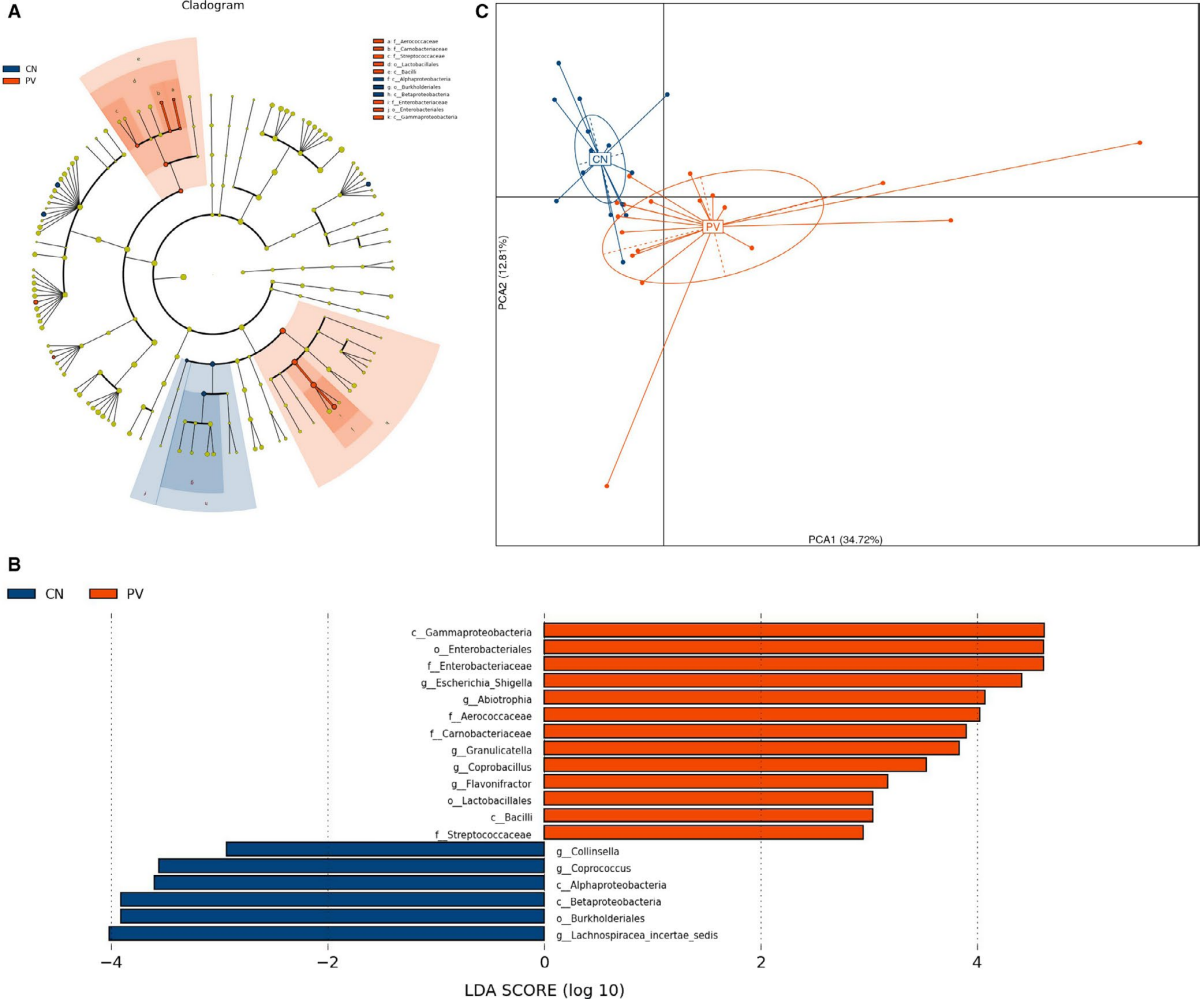
Bacterial taxa difference between PV patients and healthy controls. Different taxa were detected by LefSe (*P* < 0.05, linear discriminant analysis [LDA] >2 logs). A, Different microbes showed in Cladogram of LefSe analysis. B, Nineteen different microbes with LDA >2 logs between patients (red bars) and controls (blue bars) are displayed. C, Principle component analyses (PcoA) plot of different microbial taxa between groups

LefSe analysis was applied to investigate the significantly different bacterial taxa among groups. We found that 19 different abundant taxa existed between the PV and CN groups, and all of them had a log LDA score >2 (Figure 2A,B). Subsequently, Wilcoxon test and Kruskal‐Wallis test were performed to verify the differential abundance at the phyla, family, class, order and genera levels, respectively. Normally, bacterial taxa associated with *P* < 0.05 and FDR *q* value < 0.1 were considered to be significantly different. Although all of the FDR *q* values were > 0.1, the mean abundance of taxa between the two groups was different (*P* < 0.05), and all of the 95% CIs of mean abundance difference of these taxa never spaned 0. Finally, only 10 taxa were found to be significantly different between the two groups as assessed by Kruskal‐Wallis test (*P* < 0.05) (Table 2). At the class level, the PV group had decreased *Betaproteobacteria* and increased *Gammaproteobacteria* as compared to the control group. Both family microbes *Carnobacteriaceae* and *Enterobacteriaceae* were more abundant in patients who also showed more abundance of *Burkholderiales* and decreased *Enterobacteriales*. At the genera level, *Lachnospiracea_incertae_sedis* and *Coprococcus* were decreased, while the *Granulicatella* and *Flavonifractor* were enriched in the PV group.

**Table 2:** Relative abundance of PV and control samples. Only *P*‐values <0.05 are shown

Taxon name	MAD (CN‐PV)	Lower (95% CIs)	Upper (95% CIs)	*P*‐value
c__Bacilli	2.19 × 10^−3^	−4.79 × 10^−3^	4.04 × 10^−4^	0.019
c__Betaproteobacteria	1.65 × 10^−2^	2.78 × 10^−3^	3.08 × 10^−2^	0.028
c__Gammaproteobacteria	−8.78 × 10^−2^	−1.61 × 10^−1^	−1.45 × 10^−2^	0.034
f__Carnobacteriaceae	−4.61 × 10^−5^	−8.50 × 10^−5^	−7.11 × 10^−6^	0.041
f__Enterobacteriaceae	−8.57 × 10^−2^	−1.57 × 10^−1^	−1.48 × 10^−2^	0.012
g__Collinsella	1.11 × 10^−3^	−1.77 × 10^−3^	3.98 × 10^−3^	0.047
g__Coprococcus	5.47 × 10^−3^	−3.52 × 10^−4^	−1.13 × 10^−3^	0.015
g__Escherichia/Shigella	−5.00 × 10^−2^	−1.01 × 10^−1^	7.70 × 10^−4^	0.002
g__Flavonifractor	−2.38 × 10^−3^	−4.45 × 10^−3^	−3.11 × 10^−4^	0.012
g__Granulicatella	−4.61 × 10^−5^	−8.50 × 10^−5^	−7.11 × 10^−6^	0.041
g__Lachnospiracea_incertae_sedis	2.04 × 10^−2^	1.52 × 10^−3^	4.23 × 10^−2^	0.003
o__Burkholderiales	1.66 × 10^−2^	2.40 × 10^−3^	3.08 × 10^−2^	0.027
o__Enterobacteriales	−8.57 × 10^−2^	−1.57 × 10^−1^	−1.48 × 10^−2^	0.012
o__Lactobacillales	−2.17 × 10^−3^	−4.77 × 10^−3^	4.23 × 10^−4^	0.017

Indeed, according to the PcoA plot of different microbial taxa between groups, we could differentiate patients with PV from healthy controls using the relative abundance of 10 differential taxa at all levels (Figure 2C).

Further, we assessed the microbes in PV patients who had received treatment (PVT) and those in untreated PV patients (PVU). The results showed that with the exception of *Granulicatella*, the abundance of *Lachnospiracea_incertae_sedis*, *Coprococcus* and *Flavonifractor* genera was not different between the two subgroups (Figure S2).

### 3.4 Functional analysis

**Figure 3:**
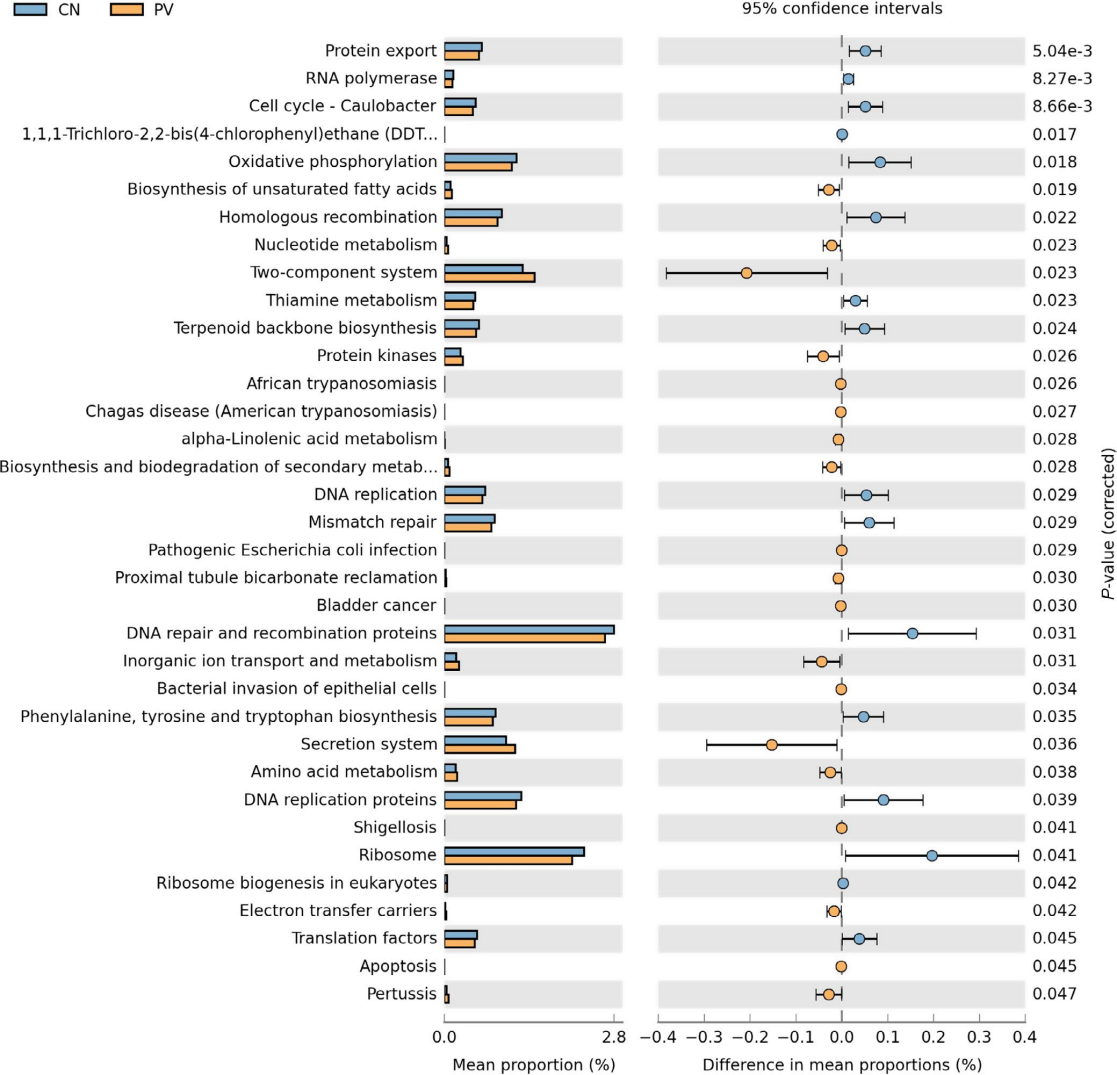
Distribution of Kyoto Encyclopedia of Genes and Genomes (KEGG) functional categories of KEGG Orthologs (KO) markers. Comparison between the healthy people‐enriched and PV patients‐enriched markers on level 3 of KEGG functional category. Abbreviation: LDA, linear discriminant analysis

Functional profiling of microbial pathways was inferred from 16S sequences with PICRUSt; we annotated the gene catalogue by KEGG metabolic modules. We found 10 and 35 differently abundant pathways at 2 and 3 levels, which suggested a diverse change in the functions of the microbiota in PN subjects when compared to controls (Figure 3A, Figure S3).

Indeed, the upregulated pathways in PV subjects were those related to metabolism, signal transduction, excretory and infectious diseases. The downregulated pathways in PV patients were those related to cell growth and death, metabolism, replica and repair, folding, sorting, degradation and translation.

### 3.5 Plasma concentrations of cytokines in PV patients and healthy controls

**Figure 4:**
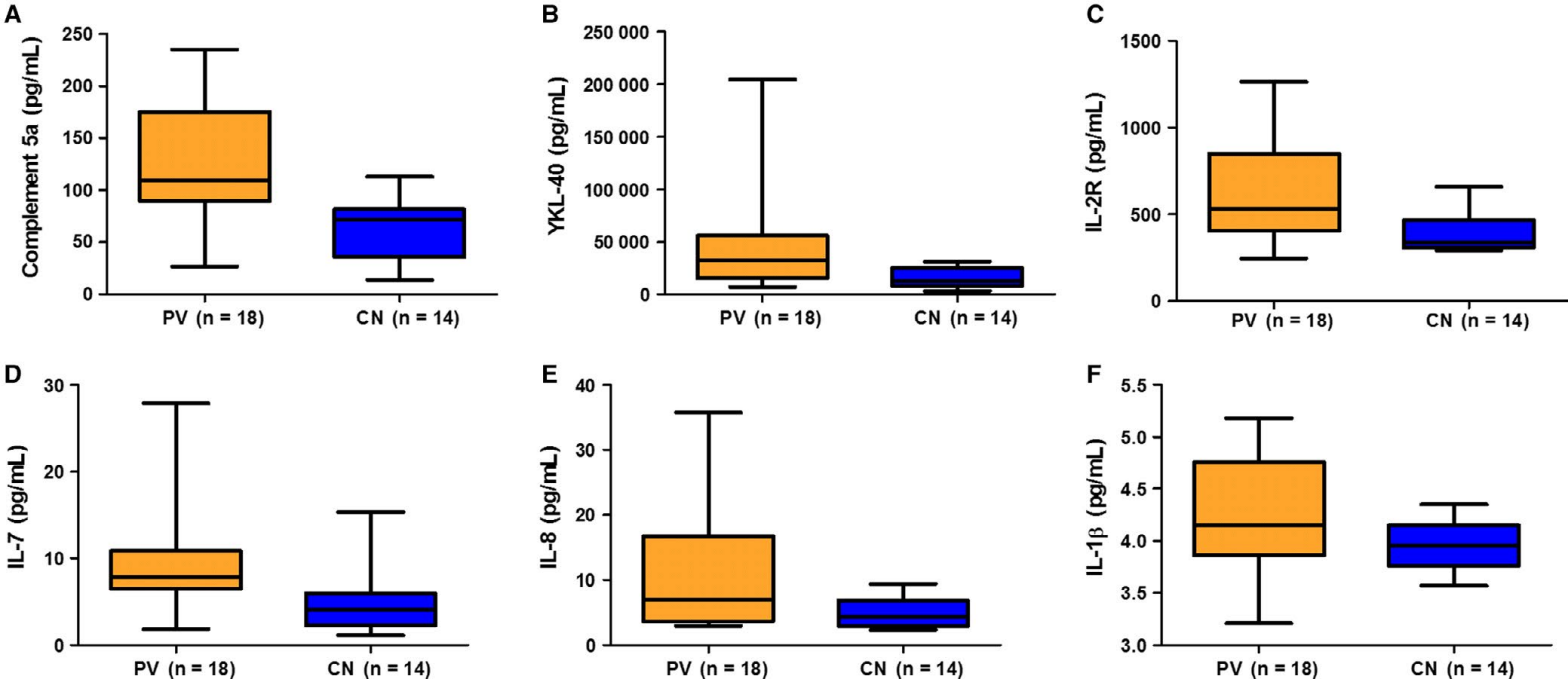
A‐F, Plasma concentrations of cytokines in patients and controls (*P* < 0.05 for all). The below and above lines indicate the minimum and maximum value. The middle lines represent the median values

Out of the 21 cytokines assessed, the levels of C5a (*P* = 0.02), YKL‐40 (*P* = 0.02), IL‐2R (*P* = 0.01), IL‐8 (*P* = 0.033), IL‐7 (*P* = 0.006) and IL‐1 beta (*P* = 0.038) in PV patients were significantly higher than those in healthy controls (Figure 4A). Additionally, plasma IL‐17A (*P* = 0.079), IL‐6 (*P* = 0.66), IL‐5 (*P* = 0.067) and IL‐21 (*P* = 0.072) showed an increasing trend in PV patients (Figure S4). Then, we compared the plasma level of cytokines between PVT and PVU groups and found that cytokines’ concentrations were not affected significantly by glucocorticoid and/or immunosuppressive therapy (Figures S5 and S6). In this study, all the patients include were screened for the presence of PV and the disordered immune and concentrations of cytokines were not inhibited effectively by such therapy, which could explain the result.

### 3.6 Correlation between plasma cytokines and microbial taxa

**Figure 5:**
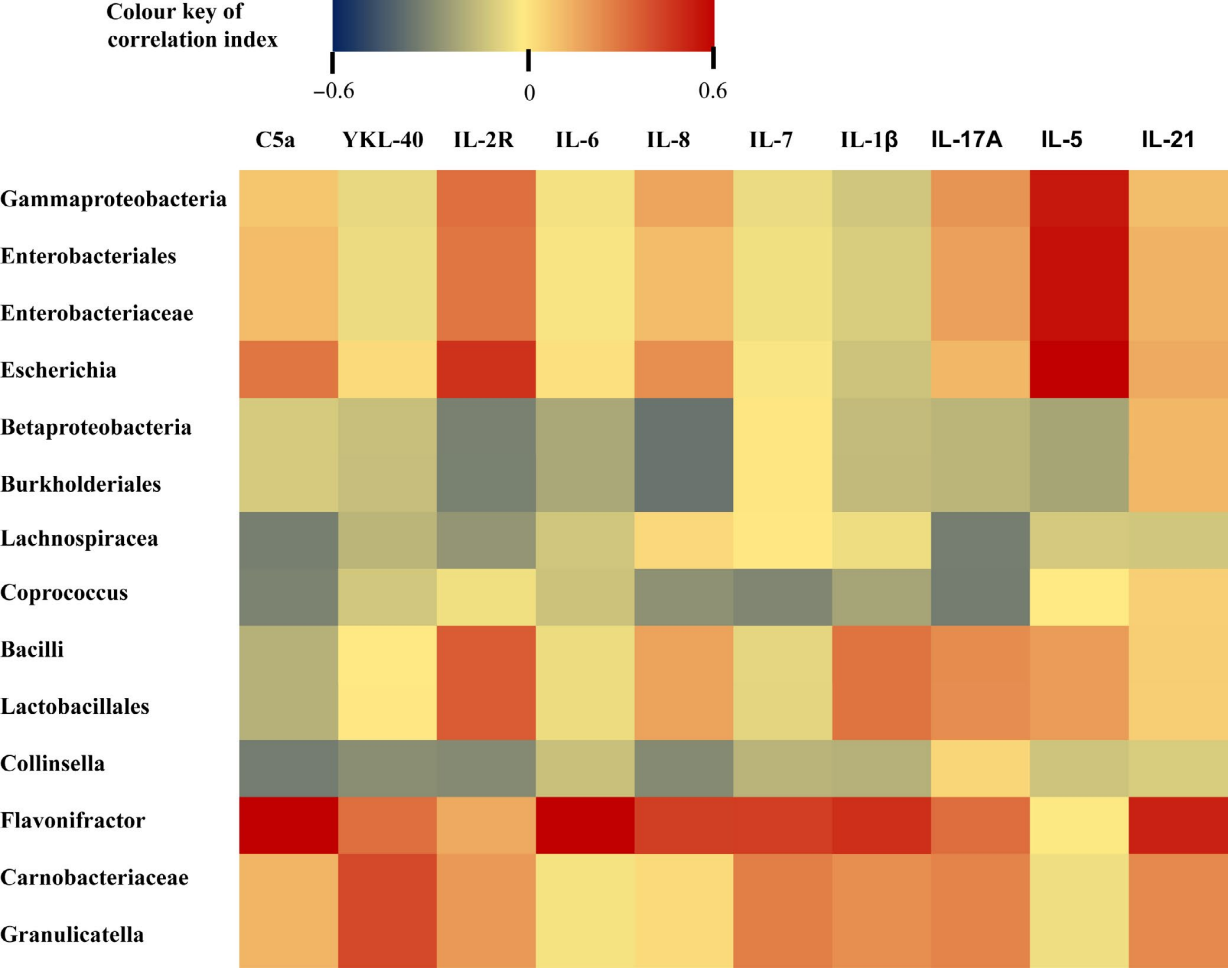
Correlation between plasma cytokines and microbial taxa was assessed by Person correlation test and displayed as a heat map

We further assessed the correlation between plasma cytokines and the gut microbiota and presented the correlation efficiency through heat map (Figure 5). Most significantly, *Flavonifractor* at genus level showed a positive correlation with C5a (R = 0.631, *P* < 0.001), IL‐6 (R = 0.814, *P* < 0.001), IL‐8 (R = 0.441, *P* = 0.013), IL‐7 (R = 0.446, *P* = 0.012), IL‐1β (*P* = 0.482, *P* = 0.006) and IL‐21 (R = 0.518, *P* = 0.003). *Lachnospiracea_incertae_sedis* (R = −0.322, *P* = 0.056) and *Coprococcus* (R = −0.325, *P* = 0.074) were both negatively associated with IL‐17A *Granulicatella* and YKL‐40 showed a positive correlation (R = 0.416, *P* = 0.002).

## 4 DISCUSSION

Metagenomic studies on gut microbiota have burst onto the scientific scene during the last decade. These studies have unraveled links between some disorders and dysbiosis in the gut microbial ecology. Remarkably, intestinal dysbiosis has also been associated with autoimmune diseases, such as rheumatoid arthritis, Behcets disease, SLE and inflammatory bowel disease (IBD).^21^, ^22^, ^23^, ^24^ Pemphigus refers to a group of IgG‐mediated autoimmune diseases; of these, a major subtype PV is associated with considerable morbidity and mortality.^25^ However, the pathogenesis of this disease remains to be fully elucidated. In addition, no studies have investigated the gut microbiota in patients with PV. This is the first study that reports the imbalance of gut microbiota in PV. At the genus level, the declined microbes were *Lachnospiracea_incertae_sedis* and *Coprococcus* and the enriched microbes were *Granulicatella* and *Flavonifractor*. In a previous study, the increment in *Lachnospiracea* abundance was reported to be consistent with microbiota changes of inhibited caspase‐1 in association with activation of pro‐inflammatory cytokines such as IL‐1β and IL‐18.^26^ Absence of *Lachnospiracea_incertae_sedis* and *Coprococcus* may cause a decline in short‐chain fatty acid production and is often associated with diseases such as asthma and inflammatory bowel disease.^27^ Additionally, decline in *Coprococcus* has also been demonstrated in patients with intestinal, neuropsychological, infectious, atopic and liver diseases.^28^, ^29^, ^30^, ^31^, ^32^ *Lachnospiracea_incertae_sedis* and *Coprococcus*, both butyrate producing bacteria, were shown to exert an anti‐inflammatory effect by inducing regulatory T cells (Tregs); consequently, these have the ability to modulate the immune system.^28^ In this study, both *Lachnospiracea_incertae_sedis* and *Coprococcus* showed a negative correlation with IL‐17A, which suggested that these two organisms may contribute to the pathogenesis of PV through regulation of T cell differentiation and the associated cytokines.

*Flavonifractor* has been shown to cleave quercetin, a flavonoid with anti‐oxidant and anti‐inflammatory properties.^33^, ^34^ Thus, presence of *Flavonifractor* may potentially, through cleaving the flavonoid C‐ring and degeneration of quercetin, induce oxidative stress and inflammation in the host.^35^ This is consistent with the observed positive correlation between *Flavonifractor* and circulating inflammatory markers (C5a, IL‐6, IL‐8, IL‐7, IL‐1β and IL‐21) in the present study. Indeed, increased *Flavonifractor* in patients with SLE, a complex autoimmune disease and patients with newly diagnosed bipolar disorder (BD) have been demonstrated.^35^, ^36^ Additionally, it was reported that specific *Bifidobacterium* strains could attenuate liver injury by downregulating the cytokines through modulating* Flavonifractor*.^28^ The other enriched gut microbe, *Granulicatella,* was originally known as a nutritional variant of *Streptococcus* and commonly reported to cause infective endocarditis.^37^ It was also shown to exhibit a positive association with severe diarrhoea.^38^ In this study, *Granulicatella* was increased in patients treated with systemic corticosteroids compared with treatment‐naive patients, which suggested its association with opportunistic infections caused by immune suppression.

T cells are mainly classified as CD4+ or CD8+ T cells. CD4+ T cells regulate antibody production by interacting with B cells and directly infiltrate the tissues expressing the target antigen, where they modulate inflammation through cytokines and surface molecules. Autoreactive CD4+ T cells have been implicated in anti‐Dsg3 antibody production.^25^ Although several recent studies have examined T cell and cytokine profiles in pemphigus patients, the results have been largely inconsistent. Overall, Th1 predominant and Th2 suppressant responses have been demonstrated in PV.^4^ Recent studies have shown that the activation of Th17 pathway may be involved in the pathogenesis of PV.^8^ In our study, we found a significant increase in the plasma concentrations of IL‐6, IL‐8, IL‐7, IL‐1β, IL17A, IL‐5 and IL‐21 in patients with PV, which is consistent with the results of previous studies. It was reported that Th cell subpopulations differentiate in gut associated lymphoid tissues and require specific gut microbes for their differentiation through the metabolites, short‐chain fatty acids.^39^, ^40^ Thus, whether the imbalanced Th1/Th2 or Th17/Tregs differentiationand abnormal cytokines in circulation were induced by changes in *Lach‐nospiracea_incertae_sedis, Coprococcus* and *Flavonifractor* in PV patients needs to be further studied.

Our study benefited from the inclusion of 18 well‐characterized patients with current presence of PV and 14 age‐ and gender‐matched healthy individuals. This is the first study that documents dysbiosis of gut microbiota in PV and also tries to explain the association between cellular immune and gut microbiota in this disease. However, some limitations of the study need to be acknowledged. First, the sample size of PV patients was small. However, this is largely attributable to the low incidence of this rare disease (range: 0.5‐50 cases per million population) 28; therefore, it is difficult to prospectively recruit a large cohort of PV patients. Second, half of our patients had started systemic corticosteroid therapy which is liable to affect the composition of gut microbiota. Nonetheless, all the included patients had current disease, which indicates that the treatment had not yet effectively induced immune suppression. Moreover, we also compared the microbes between patients undergoing treatment with systemic corticosteroids and treatment‐naive patients. Except *Granulicatella*, the abundance of *Lachnospiracea_incertae_sedis*, *Coprococcus, Flavonifractor* genus was not different between the two subgroups. This further suggests that the altered *Lachnospiracea_incertae_sedis* and *Coprococcus, Flavonifractor* in the PV group were not affected by systemic treatment.

In summary, our study employed a novel and comprehensive approach to investigate the symbiotic relationship between gut microbiota and cytokines in PV. We have identified declined *Lachnospiracea_incertae_sedis* and *Coprococcus*, and increased *Flavonifractor* in PV, and discussed its association with dyshemostasis of cytokines produced by Th1/Th2/Th17 cells. However the potential link between the gut microbiota, cytokines and PV still needs to be clarified. Further studies are required to establish a role of *Lachnospiracea_incertae_sedis* and *Coprococcus*, Flavonifractor in PV.

## 6 CONFLICT OF INTEREST

None.

## 7 AUTHOR CONTRIBUTIONS

Shuli Huang performed the experiments, collected the data and wrote the paper. Yongqiong Deng conceived and designed the study, analysed the data, interpreted the data and approved the final version of the manuscript. Xia Xiong collected the data, supervised the study and also approved the final version of the manuscript.
