# Supplementary material for: The imbalance of gut microbiota and its correlation with plasma inflammatory cytokines in pemphigus vulgaris patients
Source: Scand J Immunol. 2019 Jul 8;90(3):e12799. doi: 10.1111/sji.12799 (PMC9286422; doi:10.1111/sji.12799)
Supplement: Supplementary file 1 [file SJI-90-e12799-s001.doc]

**Supplementary material**


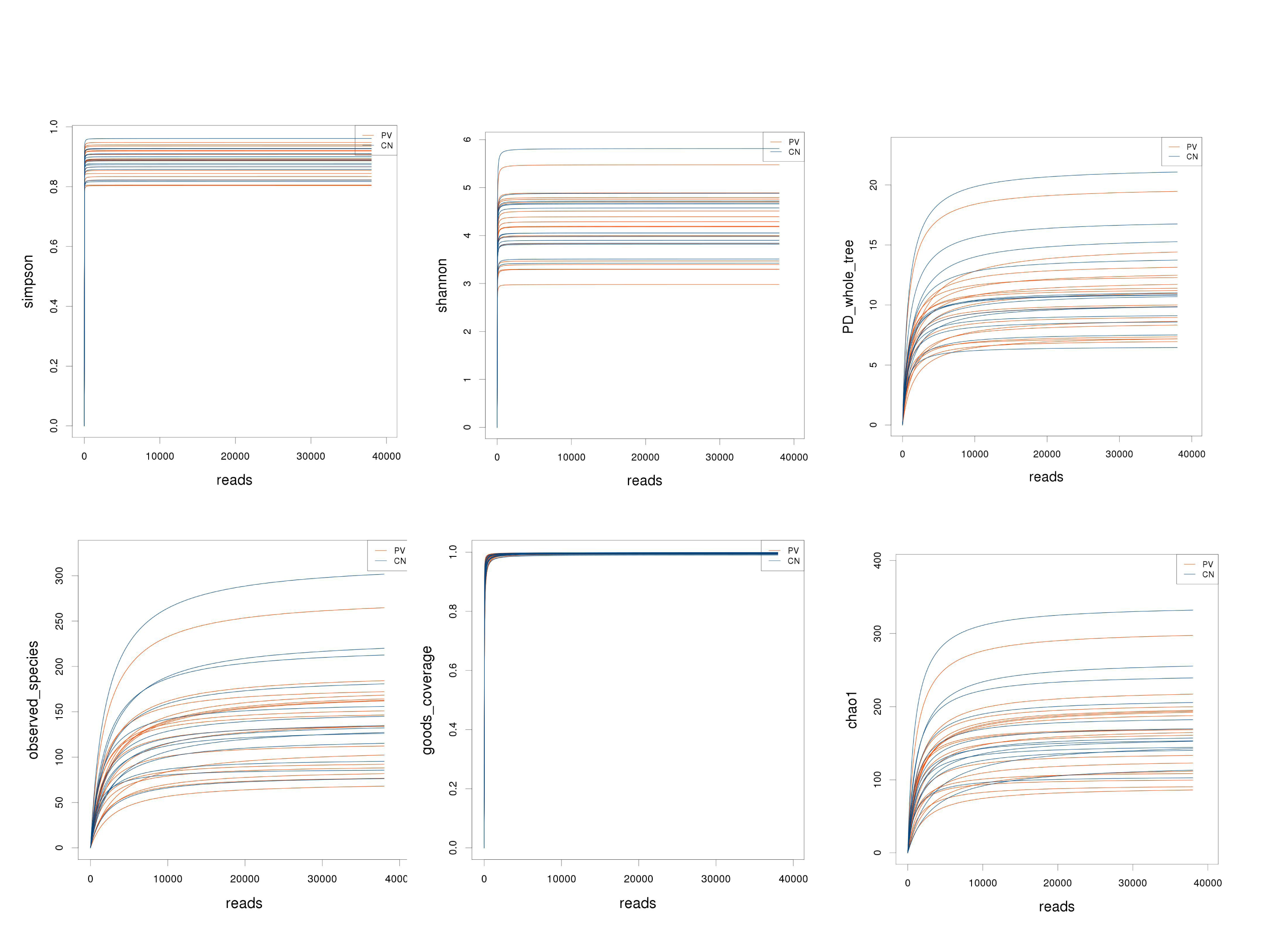


Fig. S1 Comparisons of different diversity indices in acne samples and healthy samples.


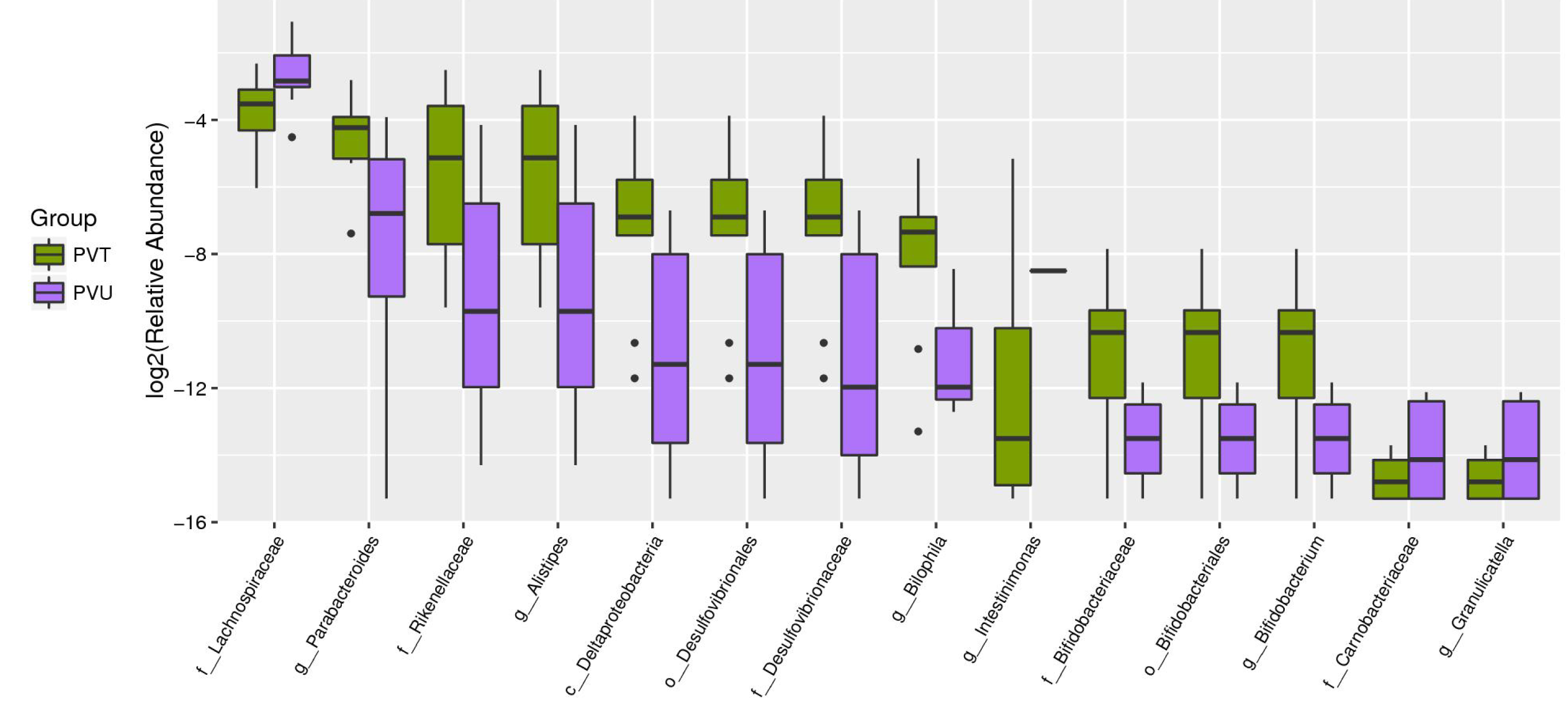


Fig. S2 Different microbes between PV patients with treatment (PVT) and untreated cases (PVU).


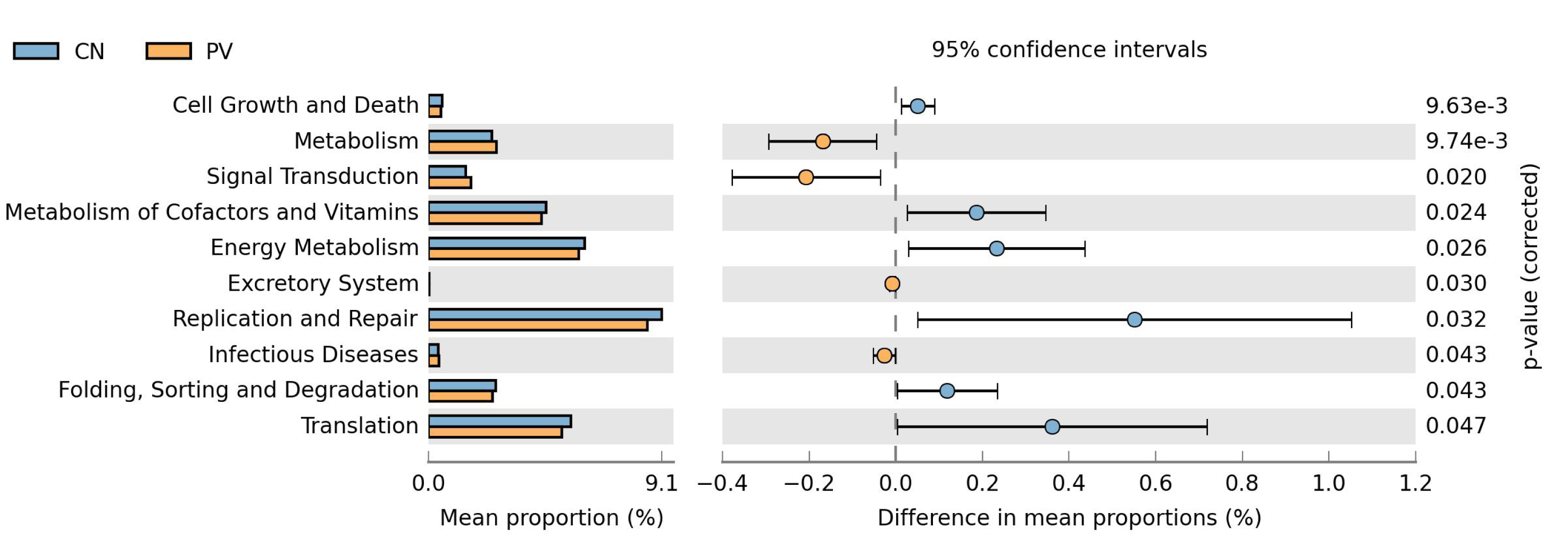
Fig. S3 Distribution of Kyoto Encyclopedia of Genes and Gemos (KEGG) functional categories of KEGG Orthologs (KO) markers. Comparison between the healthy people-enriched and acne patients-enriched markers on level 2 of KEGG functional category.





Fig.S4 (a–d) Concentration of cytokines between patients and controls, all p>0.05 and <0.1. The below and above lines indicate the minimum and maximum value. The middle line represents the medians.


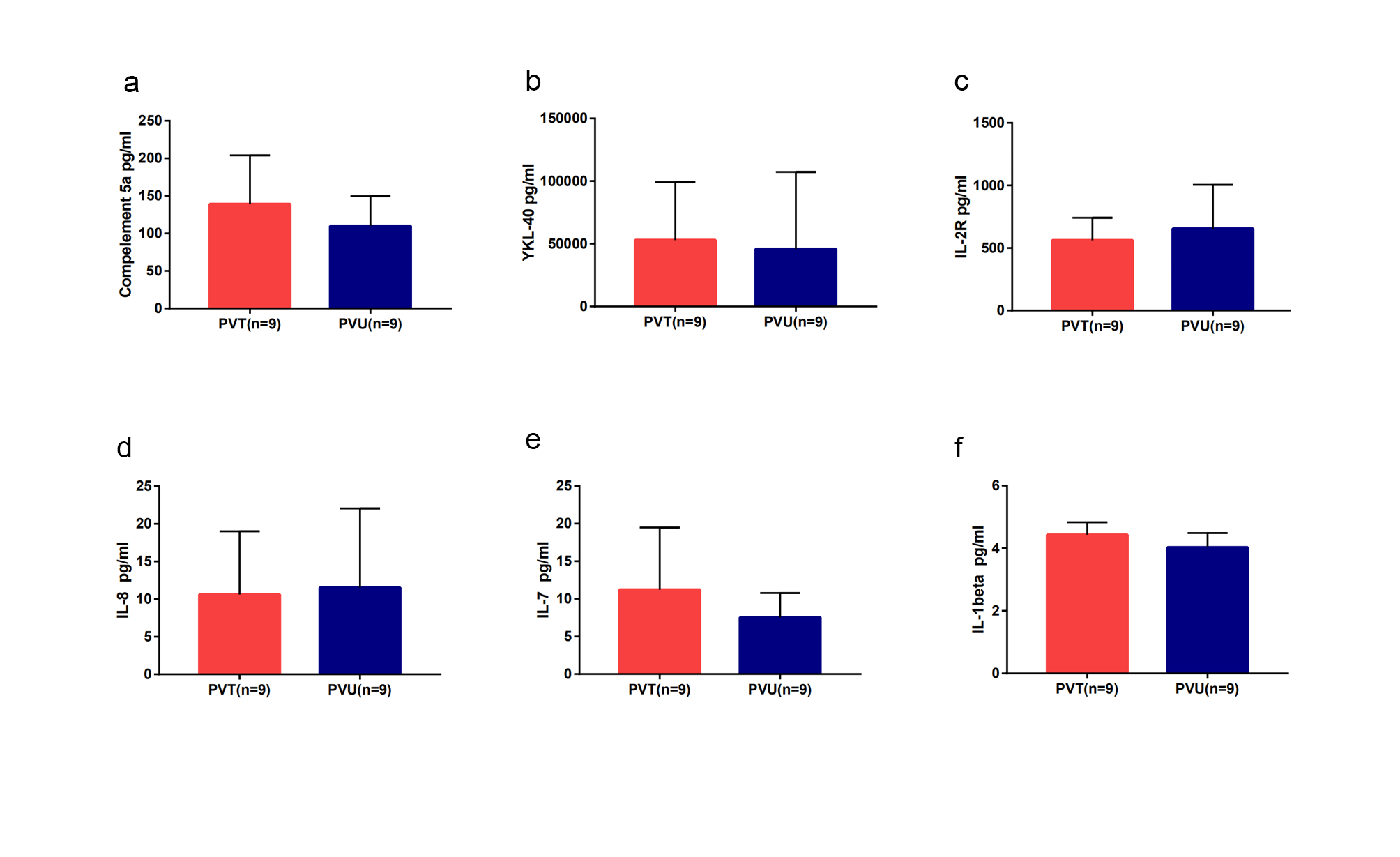


Fig.S5 (a–f) Concentration of cytokines between patients with steroid treatment and treatment naive, all p>0.05 and <0.1. The below and above lines indicate the minimum and maximum value. The middle line represents the medians.


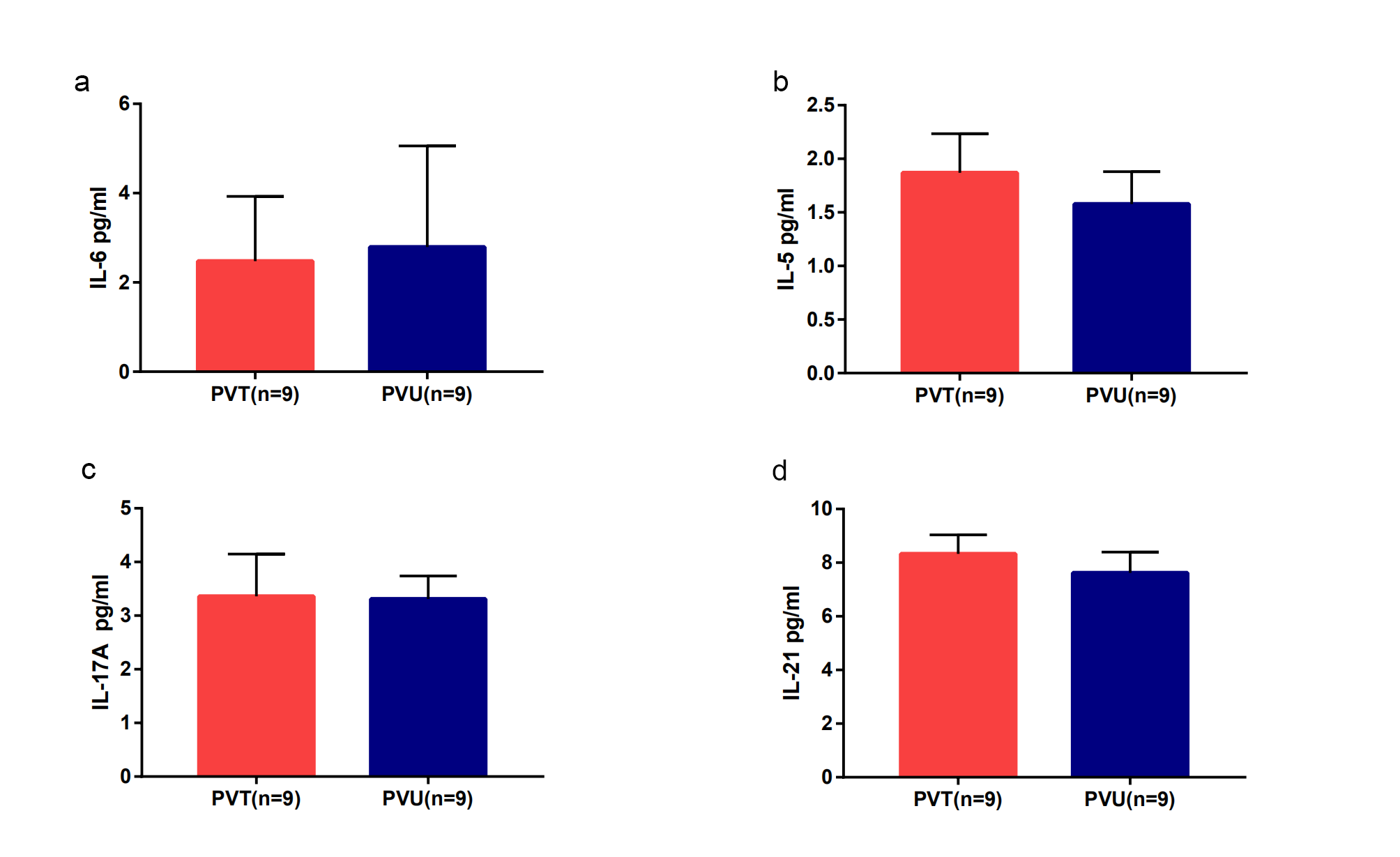


Fig.S6 (a–d) Concentration of cytokines between patients with steroid treatment and treatment naive, all p>0.05 and <0.1. The below and above lines indicate the minimum and maximum value. The middle line represents the medians.
